# Reorienting rabies research and practice: Lessons from India

**DOI:** 10.1057/s41599-019-0358-y

**Published:** 2019-12-03

**Authors:** Krithika Srinivasan, Tim Kurz, Pradeep Kuttuva, Chris Pearson

**Affiliations:** 1 Institute of Geography, University of Edinburgh, Edinburgh, United Kingdom; 2 Department of Psychology, University of Bath, England, United Kingdom; 3 Independent Researcher, Chennai, India; 4 Department of History, University of Liverpool, England, United Kingdom

## Abstract

In this article, we reflect on the institutional and everyday realities of people-street dog relations in India to develop a case for decolonised approaches to rabies and other zoonoses. Dog-mediated rabies in Asia and Africa continues be a major concern in transnational public health agendas despite extensive research and knowledge on its prevention. In India, which carries 35% of the global rabies burden and has large street dog populations, One Health-oriented dog population management programmes have been central to the control of this zoonotic disease. Yet, rabies continues to be a significant problem in the country. In this article, we address this impasse in rabies research and practice through investigations of interactions between people, policy, and street dogs. Drawing primarily on field and archival research in Chennai city, we track how street dogs are perceived by people, explore how these animals have come into interface with (public) health concerns over time, and examine the biosocial conditions that frame people-dog conflict (and thereby rabies). These analyses create a picture of the multidimensional character of people-dog relations to offer new insights on why One Health-oriented rabies initiatives have not borne out their full promise. In effect, the article makes a case for a shift in public health orientations—*away* from intervening on these animals as vectors to be managed, and *towards* enabling multispecies habitats. This, we argue, requires the decolonisation of approaches to dog-mediated rabies, and expanded conceptions of ‘healthy more-than-human publics’. In conclusion, the article chalks out broader implications for public health approaches to zoonoses in a world marked by mutual risk and vulnerability that cuts across human and nonhuman animals.

## Introduction

In this article, we bring together the public health problem of dog-mediated rabies and the concept of ‘healthy publics’ to draw out implications for research and practice on zoonoses in the contemporary world. Drawing on field and archival research in India, we build an original and grounded picture of society-dog relations to offer fresh insights on why rabies continues to be a public health concern. Our findings highlight the importance of decolonising One Health approaches to rabies. We argue for critical attention to the manners in which rabies programmes and policies, including understandings of the place of dogs, are shaped by transnational influences and power relations in the past and present. We also develop the analytics of decolonisation beyond the human, i.e., to the ‘more-than-human’, to reflect on the species hierarchies that characterise One Health approaches to rabies. Our analysis takes the concept of ‘healthy publics’ in new directions by proposing the idea of ‘healthy more-than-human publics’ for the study of disease and health at the human-animal interface.

In what follows, we use the terms people and public interchangeably to refer to ‘laypersons’ (those who do not have specific affiliations in debates around street dogs), and ‘society’ to refer to the complex of institutional arrangements and individual people with which street dogs interact. The term ‘nonhuman animal’ is used to emphasise the fact that all humans are also animals; this is in line with literatures that question the anthropocentrism that characterises much scholarship (Derrida, 2008). The terms ‘stray dog’, ‘street dog’ and ‘free-living dog’ all refer to ‘domestic dogs’ (Canis lupus familiaris) that are not owned by humans. However, the term ‘stray dog’ denotes a Western understanding of dogs as ‘out of place’ if they are not human property. We therefore follow Srinivasan (2013; 2019) in using the terms ‘street dog’ and ‘free-living dog’ to signal the legal and societal legitimacy available to ‘unowned’ dogs in India. While ‘street dog’ is the term used in Indian law to refer to unowned dogs, the term ‘free-living dogs’ reflects the fact that dogs also inhabit locations that are not built up (i.e., where there are no streets). Where necessary for analytical purposes, we use the term ‘stray dog’ to emphasise how dogs have been conceptualised in public health agendas (versus the idea of ‘street/free-living dog’ that is found in public perceptions and law in India). These different terms (stray, street, free-living) are core to our arguments. Finally, by using ‘transnational’ we foreground the possibilities for connections and differences between countries and cultures with regard to how public health issues are understood and prioritised. By contrast, the term ‘global’, when used to refer to public health agendas, projects a problematic sense of unanimity across highly varying socioeconomic and cultural contexts.

## Rabies, dogs and One Health

Rabies is a zoonosis caused by a virus that is transmitted through the saliva of infected mammals, with domestic dogs being the most common reservoir and source of transmission. It can be prevented through vaccination (pre or post-exposure), but is almost always fatal once symptoms develop. Rabies is framed as a neglected tropical disease in public health agendas, with the majority of contemporary cases (around 95%) found in Asia and Africa (Bourhy et al., 2010). In places like Europe and North America, the elimination of stray dog populations and the tight management of owned dogs through licensing, vaccination, neutering, and microchipping have been central to the control of rabies (Wang, 2012; Howell, 2015; Pearson, 2017).

Over the years, epidemiological, biomedical, veterinary, and more recently social science, research has led to substantial advances in knowledge on rabies prevention (Lembo et al., 2010; Davlin and Vonville, 2012; Sambo et al., 2014; Hampson et al., 2015; Widyastuti et al., 2015; Wallace et al., 2017; Castillo-Neyra et al., 2017; Taylor et al., 2017b; Cleaveland and Hampson, 2017; Elser et al., 2018; Degeling et al., 2018). Given the centrality of dogs to the spread of human rabies, the World Health Organization (WHO) (2019) recommends a three-pronged approach: (a) elimination of canine rabies through vaccination; (b) rabies awareness, dog bite prevention, and responsible dog ownership programmes; and (c) immunisation of people. The prevention of dog-mediated rabies is seen as an exemplary test case for the One Health paradigm (Cleaveland et al., 2014), which recognises the intersections between human and (nonhuman) animal health and emphasises interventions in animal populations in order to achieve human health (Hinchliffe, 2015; Friese and Nuyts, 2017).

One Health rabies elimination agendas have driven the formation of transnational, cross-sectoral alliances across public health, veterinary medicine and animal protection (Wallace et al., 2017; Minghui et al., 2018; Gamble et al., 2019). There has been a related shift away from ‘stray dog’ eradication as a means of controlling rabies in Asia, Africa and Latin America towards management of dog populations through anti-rabies vaccination coupled with neutering (though neutering as a rabies control strategy remains debated), and selective killing/removal (Taylor et al., 2017b; Cleaveland and Hampson, 2017).

The rationale for these programmes lies in the difficulty of eradicating free-living dog populations in porous landscapes where dog movement is difficult to control, resulting in the replacement of removed/killed dogs (Abbas et al., 2011; Taylor et al., 2017b). When dogs move from their established territories to areas vacated by the removal or killing of dogs, there is also increased chance of disease spread, aggression and bites because of population turnover and territorial behaviours. Mass antirabies vaccination (ARV) and neutering/animal birth control (ABC) programmes instead aim to establish healthy and stable dog populations as a means of preventing rabies. In principle, this One Health approach to rabies elimination also addresses cultural opposition to and ethical problems associated with the killing of dogs (Morters et al., 2013; Putra et al., 2013; Häsler et al., 2014; Byrnes et al., 2017).

Currently, mass dog vaccination (without ABC) has favour as the most cost-effective means of preventing human rabies (Wallace et al., 2017; Cleaveland and Hampson, 2017). In India, the earlier WHO-recommended strategy of ABC-ARV is mandated by legislation (since 2001) and implemented in different parts of the country (Fitzpatrick et al., 2016). An international charity is also conducting a mass dog vaccination programme in Goa (Gibson et al., 2018).

Despite an extensive knowledge-base on rabies prevention and the promise of the One Health approach, rabies continues to be a problem in many parts of the world. Scholars attribute this to inadequate policy and political prioritisation (Abbas and Kakkar, 2015; Cleaveland and Hampson, 2017; Gamble et al., 2019), implementation and resource challenges (Lembo et al., 2010; Arechiga Ceballos et al., 2014; Fahrion et al., 2017), inequitable vaccine coverage (Mani et al., 2016; Durrheim and Blumberg, 2017), surveillance problems (Taylor et al., 2017a), and lack of public awareness and context-specific strategies (Widyastuti et al., 2015; Balaram et al., 2016; Bharathy and Gunaseelan, 2017). In India, gaps between rabies research and policy/action have been identified as a key problem (Abbas and Kakkar, 2013, 2015). A wider literature has raised questions about the power imbalances and the ethical challenges that permeate the One Health approach, including “the prioritization of certain humans over other humans and other species” (Craddock and Hinchliffe, 2015; Friese and Nuyts, 2017, p. 311; Rock et al., 2017). These critiques have crystallised in the conception of ‘healthy publics’ (Hinchliffe et al., 2018). This term urges a shift in focus from seeing the public primarily as ‘targets for intervention’ and culture as a ‘barrier to efficient biomedical interventions’, and calls for the questioning of ‘what counts as healthy and unhealthy’ (Hinchliffe et al., 2018, p. 1).

In India, neither a century of state-led killing of dogs nor two decades of ABC-ARV (longer in some cities), have been successful in eliminating rabies. Public controversies over street dogs erupt every so often, and sometimes lead to extra-legal killing of dogs (Karlekar, 2008; HT, 2016). Furthermore, in spite of the scientific consensus on ABC-ARV, ongoing legal cases in Indian courts ask for the reintroduction of street dog removal/killing (Srinivasan, 2019).

In this article, we address this impasse in scholarship and practice on rabies and street dogs in India. We draw on field and archival research in Chennai and policy research covering India, and to a lesser extent, field research in Bengaluru, as explained below.

## Methods

Intervening in, and yet diverging from the substantial scholarship on rabies prevention, our research takes a step back from the focus on rabies to examine society-street dog interactions in as open-ended a manner as possible. We followed a multi-tiered research design process wherein qualitative research in Bengaluru (2010) and Chennai (2010–2015) was used to inform further mixed methods research in Chennai (2017–2018) which forms the main dataset used in this paper.

Our research in Chennai (2010 onwards; in Tamil or English, with Tamil translated into English) examined public attitudes towards and perceptions of street dogs; the characteristics of interactions between street dogs and members of the public; public knowledge about human-dog conflict (conflict here refers to negative interactions); and institutional approaches to rabies and street dogs. It included a representative sample survey; semistructured interviews with members of the public; semistructured interviews with dog-bite patients in two of Chennai’s government hospitals; and archival, observational, interview and policy research on street dog management programmes.

The survey was carried out over two days in November 2017 within the boundaries of Chennai Municipal Corporation (*n* = 401); stratification was based on gender and socio-economic status (SES). SES was determined by features of the lived/built environment, specifically, type of dwelling and access to municipal services, especially waste management, as pilot research indicated that these characteristics influence exposure to street dogs, including the nature of interactions with them. The overall response rate was 49%. The final sample had five SES categories: *Pavement dwellers*, who have high exposure and no access to services (*n* = 100); *Slum dwellers*, who have poor or no access to services, live in informal settlements, and have relatively high exposure (*n* = 50); *People in slum rehabilitation buildings*, which changes the characteristics of the built environment, including better access to services (*n* = 50); *Middle class*, who have better access to waste management services (often because of the location of dwelling in reasonably well-serviced areas), live in fully built dwellings (often with informal extensions) but in higher densities (*n* = 100); *Upper/Upper middle class*, who have regular access to municipal services and live in fully built dwellings (*n* = 101).

We used semi-structured interviews to generate from-the-ground-up data that are not restricted by researcher assumptions. The participant sample (2017–2018) included: (1) Low SES and vulnerable groups (with high potential of regular exposure and interaction) such as pavement dwellers, waste-workers, and night-time economy workers such as auto rickshaw drivers; (2) People who had registered complaints about street dogs on the national consumer complaints forum website; (3) People from wealthier socio-economic groups; (4) Key officials responsible for the ABC-ARV programme in the Chennai Corporation and employees at the ABC-ARV centres. We conducted interviews with members of the public in different parts of the city, including those that were identified through key actor interviews and pilot research as being areas of high conflict or with poor reach of the ABC-ARV programme (total of 60 interviews, including complainants on the consumer forum website, as individuals or in groups, covering 44 women and 51 men). We triangulated this data with research carried out in 2015 using similar methods (covering 49 people from upper and lower SES backgrounds and different genders) and with observational, policy, and key actor research from 2010 onward.

For the hospital-based research, we used qualitative methods to interview 30 men, 18 women and the guardians of four children in total, as well as key medical personnel (July-August 2017). The patient interviews were mostly with people from middle/low SES backgrounds, with only three patients from upper SES groups. Of these interviews, 27 were detailed (16 men, nine women, and guardians of two children) while the remainder gathered information such as source and location of bite. In addition, we draw on research in Bengaluru which was carried out in 2010; it involved semi-structured interviews with key actors and members of the public, and discourse analysis of debates around street dogs in the city after the mauling of two children in 2007 (Srinivasan, 2015).

The survey data were analysed using primarily descriptive statistical techniques, with inferential techniques (ANOVAs) used to test for statistically significant differences between demographic groups. The qualitative data were analysed using open, axial, and selective coding techniques. Initial themes were derived through careful reading of the datasets, following which themes were categorised into umbrella codes and/or sub-themes. We then cross-checked these codes and themes against the data, triangulated them across datasets and among team members, and discussed them with reference to relevant literatures, to build analytical concepts and lines of argument (Corbin and Strauss, 2008).

Throughout the research, we deployed methods and instruments geared towards enabling participant-led insights. Many survey questions allowed participants to generate free responses instead of providing a list of options, while the semi-structured interviews and observation protocols were based on broad themes. To our knowledge, this is the first sustained and in-depth empirical study of society-street dog relationships in India that examines social, multispecies, institutional, and public health dimensions simultaneously and in an open-ended manner that reaches beyond a singular focus on rabies and/or dog bites. Through analyses of these datasets, and in conversation with recent scholarship on healthy publics (Hinchliffe et al., 2018) and more-than-human geographies (Blue, 2015; Degeling et al., 2018), we develop a grounded understanding of the wider context within which rabies emerges and is addressed. Our aim is to generate new insights on the persistence of rabies as a major public health concern despite current scientific consensus on how it can be eradicated, and more than a century of state-led dog control in India.

## People and street dogs in Chennai

In this section, we reflect on key aspects of our research to track the everyday realities of people-dog relations, explore how these animals intersect with (public) health concerns and programmes, and understand the conditions within which people-dog conflict (and rabies) materialises.

### Attitudes and perceptions

One of the main questions we sought to address was that of how people perceive street dogs. Through the survey, we asked eight questions on attitudes towards street dogs, which captured the extent to which respondents have a perception of street dogs as:

- animals they dislike
- animals they are fearful of
- a problem in Chennai
- a pest
- a nuisance
- not belonging in Chennai
- not having a right to live on the streets
- not being ‘*paavam’* (harmless/vulnerable)

These items were scored from 1 to 5 with higher scores representing more negative attitudes towards dogs (3 representing the neutral scale midpoint) (Supplementary Table 1). The responses to these items were averaged to form a composite measure, the Attitudes Towards Street Dogs (ATSD) scale. ATSD scores for the sample displayed a relatively normal distribution around a mean of 3.07 (standard deviation of 0.608). Of all the respondents, 37% had overall scores falling below the mid-point, 56% above the midpoint, and 7% exactly on the scale mid-point.

An ANOVA was conducted to assess the effect of participant gender and SES (and their interaction) on their attitudes to street dogs. This revealed no gender differences in ATSD (both in and of itself, and through interaction with socio-economic status)^1^. However, we did observe a statistically significant effect of socioeconomic status (SES) on ATSD (F(4, 391) = 2.77; *p* = 0.03). People in Slum Resettlement buildings had the *most negative* attitudes to street dogs (mean ATSD (M) = 3.21; Standard Error (SE) = 0.085). The next most negative group was Upper Income (M = 3.138; SE = 0.06), followed by Pavement Dwellers (M = 3.12; SE = 0.06), Slum Dwellers (M = 3.049; SE = 0.09), with the Middle Income group showing the most positive attitudes (M = 2.92; SE = 0.06). These results show that, surprisingly, there is no *straightforward linear relationship* between SES and ATSD. The poor are at the greatest risk of dog bites and rabies (World Health Organization, 2018a). One would therefore expect attitudes to be the most negative in the lower end of the SES scale. However, this is not borne out by the survey data.

More interestingly, a majority agreed or strongly agreed that street dogs were a problem (71.6%; 95% confidence interval ±4.41^2^), a pest (70.6%; ±4.46) and a nuisance (69.3%; ±4.51). Nonetheless, a majority also believed that street dogs have a right to live on the streets (78.8%; ±4.0), that they belong in Chennai (55.5%; ±4.86), and that they were *‘paavam’*/vulnerable creatures (79.3%; ±3.96)^3^. The semi-structured interviews revealed a similar complex of conflicting attitudes within *individual* participants. Most interviews would begin with an elaboration of either positive or negative views on dogs, but over time, would evolve to a discussion of other viewpoints.

For instance, Thamarai (F/LIG/2017)^4^ started with complaints: “There are too many street dogs here…In the night if dogs bark, I am scared to go out”, but at a later point in the interview, talked about how “we put the leftovers on the street. And these dogs eat that food. I do not feel like wasting it. Instead, I feel that it is better these dogs, Brownie, Blackie, eat it.” By contrast, Bala (M/MIG/2017) initially said that “I do not see them [street dogs] as a problem…dogs are companions of Bairavar [a deity],” but at a later point: “They [dogs with mange] will be sick. You would feel nauseated looking at them…If you ask me, they should catch those dogs and kill them.”

In essence, public perceptions of street dogs are more complex than either positive or negative. Street dogs are seen as posing risks or as nuisances. Yet, there is a recognition of these animals as vulnerable creatures that belong in the city even though they are not ‘owned’ by people (cf. Srinivasan, 2019). Referring to the city itself as their (street dogs) home, Sarasu (F/LIG/2017) explains: “Everything here is their house…they are living in their house only…They are also living beings, right?” These animals are seen as rightful cohabitants even though they might pose problems. As Chandran (M/UIG/2017) put it: “I do not think street dogs are particularly beneficial or particularly problematic. They have been a part of our society. You cannot classify people as beneficial/problematic, same way.”

This is not to say that all interviewees felt that dogs belong in public spaces. In the semi-structured interviews, some upper SES participants expressed the view that dogs do not belong in the city. They also drew comparisons between street dogs and people who live and work on the streets, such as street vendors or pavement dwellers, mirroring what Baviskar (2011) has referred to as bourgeois environmentalism in the context of the urban elite in India’s capital city, Delhi. To Anu (F/UIG/2017), “dogs should not be left on the streets. Let us also allow people to live on the streets then. There are so many homeless people, they can also come in and sleep on the street. Where do you draw the line?” Shiva (M/UIG/2017) held similar views: “One of the primary reason for the street dogs on the beach are the *bajji* [a snack] sellers. They feed the dogs…Even these *bajji* sellers should be removed from the beach, not just for curbing the street dog problem, but for improving the aesthetics of the beach.” And to Hariharan (M/UIG/2017), “previously, in olden times or rural areas, they [street dogs] might be useful to scare away thieves and burglars. But, here, now in the city, they are not needed for that purpose. What is the purpose of that dog on the street? I am not against animals…let people have them as pets, control them, keep them chained, take care of them.” In this view, dogs need to serve a (human) purpose in order to be permitted to exist.

In effect, the idea that street dogs are out of place in modern cities filters into people’s narratives, especially among higher SES groups. The survey finding that the slum resettlement group has the most negative attitudes towards street dogs also raises the question of whether the move from informal settlements to fully constructed buildings is linked to shifting worldviews about what urban life ought to be like, i.e., shifts from an everyday acceptance of street dogs as part of the neighbourhood to more sanitised visions of urbanity. These shifting normative ideas about modern cities exist alongside long histories of human-street dog cohabitation and ideas of dogs as rightfully belonging in public spaces.

### Conflict and care

Another focus of our research was interactions (positive and negative) between people and street dogs. Survey participants were asked to list what they perceived as problems linked to street dogs. The most common complaint (or perceived problem) was barking (reported by 53.9%; ±4.88), followed by chasing (50.1%; ±4.89) and biting (39.2%; ±4.78). Other complaints related to infections, dirt, dogs causing accidents, aesthetics, fear of dogs, threats to pets, and as dogs not being suitable in a developed country^5^. Only 15% (±3.49) of survey participants mentioned rabies as a problem—a lower percentage than those who found dogs to be ‘ugly’ (22.9%; ±4.11)—though it is possible that those who mentioned ‘biting’ as a problem had rabies in mind. There were some survey participants (5.1% (±2.15)) who said that they did not associate street dogs with any problems.

The semi-structured interviews generated similar complaints, and indicated that dogs that triggered complaints tend to be associated with specific people. Babu (M/Pavement dweller/2017) explains: “When I eat, these *vayilla jeevans* [voiceless creatures] come stand next to me. So, I feed them what is left over after I eat… All these dogs started to stay right here itself with me … But some people ask me, why do you have so many dogs? They complain that my dogs bark.” Similarly, Krupakaran (M/MIG/Policeman/2017) attributes dog-related problems to a local autorickshaw driver: “Kids have been chased by these dogs. They are fed by that auto-rickshaw driver there. He himself does not have food. He goes begging and gets food that he feeds to these dogs.” This raises two questions for further research: (1) are street dogs with strong attachments to people more likely to display territorial behaviours (as pet dogs do)? (2) are people-dog conflicts tied to conflicts between people?

We asked people if they had ever intentionally hurt street dogs. Some, 20.69% (±3.96), reported having hit or thrown stones at street dogs. By contrast, 64% (±4.7) indicated that they had offered food or water to a street dog at least once and 19% (±3.84) indicated that they had taken regular care of a street dog.

Semi-structured interviews with pavement dwellers and waste-workers indicated strong relationships with specific street dogs. People from low-income backgrounds, especially pavement dwellers, tended to see dogs as sources of comfort: “We give them *porai* [hard bread], biscuits. So they get accustomed to us…We interact, no? (*pazhagurom*, ille?) That dog plays with us with love, no? Sleeps next to us. After we are asleep, they sleep on our legs” (Karuppiah/Pavement Dweller/ 2017). Street dogs were also seen as enabling a safe environment. As Komala (F/Pavement Dweller/2017) said: “See, sometimes drunkards who pass by in the night, they might sometimes try to come sit next to me. Even sleep next to me. They might try to steal, if there is some money….If this fellow [referring to a dog] is nearby, he starts barking…”

These interviews indicated that casual feeding of dogs with scraps was very common. All the waste-workers and pavement dwellers interviewed regularly fed street dogs with leftovers, biscuits, or in the case of waste-workers, by separating food from the waste they sort through. “I give them whatever food I find in these bins. Sometimes you get food, sometimes you do not. When you do not get, there will be dogs looking at you longingly for food…*Romba kashtama irukkum* [it makes you feel very bad]” (Velu/M/Waste-worker/2017). They were able to identify individual dogs in their areas by colour, behavioural characteristics, and often, name. “One brown guy usually comes near this particular dumpster and waits for me to give him something…One black and white spotted dog barks quite vigorously at me here. That fellow would get on top of the vehicle and bark. He never bites or comes near, just barks from a distance and goes away. I know most dogs in this neighbourhood” (Shanmugam/M/Waste-worker/2017). Another waste-worker, Ramu (M/19/4/17), names the local dogs: “Rendu Moonji, Karuppa, Samy, Sevulu, Tiger.”

Waste-worker interviewees said that the introduction of tall, metal waste bins had reduced street dogs’ access to waste food, making them more dependent on the help of waste-workers to secure food: “The feeding of dogs started when metal bins were introduced. Because dogs could not get inside and eat. So, if we come, they will look at us. Yeah, thinking I might put something. He will come close, stand and look, plead” (Raghu/M/Waste-worker/2017). Considered altogether, what these interviews highlight is that people-street dog relationships are multidimensional; they encompass a range of positive and negative interactions that affect both people and dogs.

### Knowledge

We sought to find out through open-ended survey questions what people knew about avoiding conflict (being bitten or chased). On being invited to indicate how they thought one could best avoid being bitten or chased, the response most commonly offered (by 48% of the sample; ±4.89) pertained to ‘shouting’ at the animal, followed by ‘walking slowly away’ (39%; ±4.77), ‘throwing stones’ (37%; ±4.73), and ‘stand still and look away’ (26%; ±4.29). Some survey participants (24% (±4.18)) said they would do ‘nothing’, while 12% (±3.18) said that they would ‘run away’. A small proportion said they would befriend the dog (5%; ±2.13), ‘give it food’ (13%; ±3.29), or ‘talk to it’ (3%; ±1.67), while 0.7% said that they ‘did not know’ what to do, and 4% (±1.92) said that they would ‘avoid eye contact’ with the animal. These responses indicate a mixed situation with regard to knowledge about avoiding conflict (for e.g., ‘running away’ is not advised (Boston Public Health Commission, 2019; WHO et al., 2019). We also asked what people would do if chased by a dog while on bicycles, scooters or motorbikes (Supplementary Table 2). Other than ‘stop the bike’ (41%; ±4.81), none of the responses reflected appropriate knowledge about how to prevent chasing as they are either ineffectual or have the potential to cause accidents. On the whole, these responses point to the need for awareness programmes aimed at preventing these types of conflict and that are tailored to the specific socio-environmental contexts within which people-street dog interactions unfold.

The semi-structured interviews (Table 1), especially those with pavement dwellers, waste-workers and night-time economy workers, generated a wealth of knowledge on how to prevent conflict, which appears to stem from close everyday attention to the ecologies and cultures of the dogs. In the words of Ramu (M/Waste-worker/2017): “You should not get scared [*padaravey koodathu*]. You should not run or make any sudden movements. They will come very close, but they will not bite. Be casual…they will go away. They will keep barking but after a while they will stop…Talk to them. Say things like ‘what do you want?’ Or gently say, ‘keep quiet’. Next time make sure you feed them something. But, first, you have to be casual with dogs. Do not fear them at all.”

Survey participants’ free-responses to being asked what causes aggression in dogs indicated familiarity with key triggers. As shown in Table 2, only 10% of respondents said that it was in the nature of dogs to be aggressive.

Most of the hospital-based interviews also indicated insight (among patients) on the circumstances that had led to their being bitten, and the general view that the dogs were not to ‘blame’. Some examples:

“One cannot blame the dog…It was a female with pups…The dog was just trying to protect its pups and I went too close and it bit me (Abdul/M/MIG/2017).

“I was talking on the phone with my boss and did not notice the dog sleeping on the street…I stepped on it, not once, but twice. The dog turned around and bit me. I feel sorry for the dog, as I think I hurt it. The dog is still limping. Others wanted to kill it. I stopped them. What did it do? It was not its fault. I was the one who did not notice…If I go talking on the phone and not notice a car, and I get hit, I am the one to blame, right?” (Harsh/M/MIG/2017).

With regard to post-exposure prophylaxis, 81% (±3.84) of survey participants said that they would seek medical treatment but some responses (such as seeking magico-religious treatment) indicate that there is a need for improved awareness (Supplementary Table 3). In the hospital-based research, the importance of washing wounds with water (and soap) as a first aid measure was mentioned by most patients, and by default, all of them had sought medical attention. However, knowledge among bite patients about *preventing* dog bites was rather weak. People talked about staying away from unknown dogs, generally being ‘more careful’, avoiding dogs, avoiding dogs with puppies, vaccinating pet dogs, and street dog eradication/confinement. While none of these are ‘incorrect’ answers, they nonetheless point to the need for context-specific education on how to live safely with street dogs. This is especially important given that some patients had had prior experiences of being bitten.

### The ABC-ARV programme

While Chennai has a well-established and lauded ABC-ARV programme (Krishna, 2010a; Abbas et al., 2011), our research raises issues that need further investigation. First, we found that the implementation of the ABC-ARV programme is based on administrative boundaries and does not take into account the recommended 70% coverage (of dog population) (Taylor et al., 2017b). While the municipal authority is responsible for the capture of dogs, the ABC-ARV centres are run by animal welfare organisations or by the municipal corporation with veterinary doctors employed on contract. Programme implementation is typically on a complaint basis, with no consideration of dog territoriality and the biophysical boundaries that restrict dog movement.

Cleaning and support staff reported having to work with inadequate infrastructure, and in overcrowded kennels without appropriate equipment, training and protective gear. This has consequences for not only the efficacy of the programme and staff morale and wellbeing, but also the welfare of the animals. Veterinary doctors are paid poorly for their services (Indian Rupees 100 per surgery as on 25/10/17), which has implications for the experience and competence that can be secured. Our observational research suggests that the implementation of the ABC programme goes along with serious welfare implications for the dogs at all stages, from catching, to surgery, recovery and release. Common problems include injury and mortality during capture/transport and after surgery; dehiscence (where the surgical incision opens); dehydration; post-operative and nosocomial infections; rough and incorrect handling; overcrowded and unhygienic kennels; low-quality and insufficient feeding; and delayed or incorrect release (also see Nolan, 2006).

These problems exist even though animal welfare organisations are involved in implementation, and are the most acute in ABC centres that have no animal welfare oversight, as detailed in a recent report commissioned by the Madras High Court (Advocate Commissioner et al., 2019). This report emphasises that the implementation of the ABC programme is not in line with the standard operating procedures laid out by the Animal Welfare Board of India, and outlines multiple significant concerns, including in relation to veterinary integrity, noting that “conducting so many surgeries in one single day raises serious questions about the quality of care afforded and surgery conducted” (Advocate Commissioner et al., 2019, p. VI). There is a noticeable paucity of research on the welfare implications of ABC-ARV programmes, and this is an area that requires urgent enquiry.

## Decolonising rabies

Abbas and Kakkar (2015) attribute the continued prevalence of rabies as a public health concern in India to a research-policy disconnect. While acknowledging the value of this argument, our research indicates that this impasse might be equally rooted in public health paradigms that are (neo)colonial in their understandings of what constitutes healthy societies, how to achieve them, and what resembles appropriate relationships between people and dogs.

During the nineteenth century, Western practices and norms about dogs crystallised around dog breeds and pet-keeping. ‘Unowned’ dogs were problematised as ‘stray’, with canine mobility and reproduction heavily restricted (Howell, 2015). While the Western model of dog ownership was—and is—far from universal, over time, and with colonial and postcolonial flows of ideas, policy, programmes and resources, it has become established as the normative pinnacle globally. Although free-living dogs comprise the majority of the global population of dogs, they are now often seen as deficient animals that fail to live up to the Westernised norm of dog-keeping (Coppinger and Coppinger, 2016). Public health (and animal welfare) organisations may advise against killing street dogs, but still treat them as populations that need surveillance and management, promoting ‘responsible dog ownership’ as a key foundation for human and animal health (Taylor et al., 2017b; World Health Organization, 2018b).

As discussed, scientific consensus on the prevention of dog-mediated rabies has shifted from killing/removal to One Health-oriented vaccination, neutering, awareness and responsible dog ownership programmes. On the surface, rabies eradication through killing (of dogs) appears to be fundamentally different to One Health approaches. These two strategies, however, are underlain by the common colonial (and anthropocentric) conceptualisation of free-living or street dogs as ‘stray’ animals and disease vectors to be managed for human health. That, of course, is not to say that colonial and One Health approaches are synonymous. Rather, more attention needs to be paid to the cultural, historical and transnational aspects of the links between health, humans and dogs in India. Currently, any lack of success in dog/rabies control strategies (killing/ABC-ARV) is commonly attributed to implementation problems, i.e., to deficits in resources, political will, public participation etc. While these are valid points, we argue for the consideration of more deep-seated historical and socio-cultural issues.

### The place of dogs

The issues start with fundamental differences in understandings of the place of free-living dogs in human society. Current public health approaches (whether killing or ABC-ARV) are based on (neo)colonial views of free-living dogs as ‘stray’. Even when rabies research and practice appears to originate from India, it is still produced within a Western epistemological climate and replicates Western conceptions of dogs as having to be under human ownership (Rock and Degeling, 2013). These transnational influences date back to colonial India where the British introduced state-sponsored dog eradication programmes (Krishna, 2010b).

In nineteenth century Britain, continental Europe and the United States, the growing condemnation of animal *and* human ‘vagabondage’ hardened attitudes towards ‘unowned’ dogs who were increasingly treated as symbols of uncivilised urban cultures and blamed for spreading rabies (Pearson, 2017). These attitudes and policies were imported to India, where British authorities treated street dogs as part of the problematic Indian urban environment that needed controlling and containing. The British were aware that the killing of dogs was unpopular, especially after riots in Bombay in 1832 following such killing (Palsetia, 2001). But they carried on anyway, and dogs became enfolded within the complexities of public health in British India. In 1879, dogs straying the streets of Chennai/Madras were killed under the orders of the Commissioner of Police, purportedly to combat *‘hydrophobia and other loathsome diseases’* (Health Department, 1880). The 1913 Administration report of the Corporation of Madras Health Department indicates that in that year 3003 male dogs, 2957 female dogs and 392 pups were killed (Health Department, 1914)^6^.

While it is important not to simplistically blame colonial-era policies for contemporary problems, closer research is required on the history of dogs in colonial India and the particular ways in which ideas and practices from Britain (and continental Europe and North America) have influenced dog management policies before and after independence. What is clear, however, is that transnational influences shape networks, initiatives and resource flows around rabies and street dogs (Srinivasan, 2013; Minghui et al., 2018). Furthermore, colonial and (neo)colonial legacies impact, directly and indirectly, public health agendas towards dogs, as they do in other health, development and environmental spheres in India and elsewhere (Fairhead and Leach, 1996; Amrith, 2006; Li, 2007; Ax et al., 2011; Pearson, 2018).

In reframing mainstream rabies research and policy as (neo) colonial, we are *not* claiming that such research and policy serve vested interests or are based on nefarious motivations. To the contrary, there is no doubt that the wealth of knowledge and programmes on rabies stem from well-intentioned efforts to achieve public health. Our point rather is that dogs occupy quite a different place in Indian society (even if this constantly evolves under the influence of transnational flows of ideas and practices). As the Tamil term *‘theru nai’* (street dog) conveys, these animals are seen as rightfully belonging in public spaces—dogs that are not under human ownership are not automatically ‘stray’. A 2001 change in Indian law dismantled the colonial categorisation of free-living dogs as ‘stray’ (in the Prevention of Cruelty to Animals Act introduced during British rule), and reinstituted in law the local understanding of free-living dogs as ‘street dogs’ (Government of India, 2001). This piece of legislation nonetheless contains transnational influences in that it replaces state-led control through killing with ABC-ARV.

This culturally-specific but colonially-inflected understanding of the place of street dogs is reflected in the seeming contradictions in public attitudes towards these animals discussed in the earlier section. People see street dogs as nuisances or as posing health risks. But these animals are also seen as belonging in public places: “There would be about 100–200 dogs in this neighbourhood… Only if we bother them, they bother us….These dogs live along with people, like people (*makkaloda makkala vazhuthu*)” (Maheshwari/F/LIG/2017).

The ‘public’ here is multispecies, ‘more-than-human’ (Blue, 2015). Dogs are perceived as *‘paavam’*, and as *‘jeevan’*—as vulnerable living beings who are susceptible to various harms and suffering, and who are a part of society. This is reflected in fluctuating responses to high-profile dog culling events, as occurred in the city of Bengaluru in 2007. Public protests asking for tighter street dog control after the deaths of two children by mauling morphed into protests *against* the rounding up and (extra-legal) killing of street dogs by the municipal authority (Karlekar, 2008). Similar trends can be seen in other culling events around the country (HT, 2015).

One might interpret narratives about dogs as vulnerable or as belonging in the city’s public spaces as outcomes of a certain lack of choice (especially among lower-income communities) or as manifestations of upper/middle-class post-materialist values about animal welfare. However, as both the survey and the qualitative research show, these narratives cut across SES groups and manifest in everyday practices of care. Our research (in 2010) in a low-income multi-religious community in Bengaluru, where a young girl had been killed by dogs in 2007, corroborates these findings: community members had not developed any long-term antipathy to street dogs even though they very clearly remembered the incident. Street dogs were to be found all over the neighbourhood. Community members spoke about setting aside leftover food for them, shared stories about a young boy who was famous for his close interactions with the local street dogs, and reassured the researcher that they had nothing to fear from local dogs that barked at them. Given the high-profile character of the 2007 mauling and the protests that followed (The Hindu, 2007), this community could have garnered the administrative and political support necessary to keep their neighbourhood free of street dogs if they had wished to. Indeed, they had been successful in getting a row of meat shops in that area removed as they attributed the mauling to dogs fighting over meat waste near these shops. They had, however, not actively pursued the option of excluding street dogs from their neighbourhood.

We thus argue that it is useful to not dismiss people’s narratives (and associated practices such as feeding) about canine vulnerability and belonging as born out of lack of choice or awareness. The task of decolonising rabies research and practice starts with taking these ideas about the multispecies and more-than-human character of the public—and healthy publics— seriously, and examining how they might reconfigure public health agendas.

### The multidimensionality of dog-related public health

Our research also highlights differences in understandings of the intersections between dogs and public health. Current public health agendas prioritise *rabies* as the principal health risk posed by dogs. Our research however suggests that rabies features rather low in people’s hierarchy of concerns regarding street dogs. The obvious interpretation of this finding would be to say that this is because of lack of awareness.

Moving away from this ‘deficit’ interpretation, we ask what it might mean to give serious consideration to public views of the links between dogs and public health. Issues such as barking, chasing and even just ‘nuisance’ appear much more salient risks to people than does rabies (also see Rock et al., 2017). Furthermore, people-dog conflict is not only about rabies, bites and chasing, but also about dogs being attacked or killed by people (Nath, 2016): there is *dog-on-human* conflict to be sure, but there is also *human-on-dog* conflict. Even more important, as discussed earlier (Section ‘Conflict and care’), street dogs feature in *positive* ways in what people say and do: they are seen as companions, as providing security, as subjects of affection and care.

To Aparna (F/MIG/2017), “They are on the street, but someone takes care of each of them. This lady Rosie is ours, meaning I take care of her. But she is always outside. Then, there is Tommy who is sitting near the pump. He’s another person’s… there are about 5–6 dogs in this part of the street. If you go further inside, there are some more. We all give them food: Meat, fish curry, egg…that biriyani shop man in the corner feeds them. There is always water here. Next to the pump, in front of houses. We see them as family members.” Such interactions of care indicate that street dogs are valued members of society, and that the role that positive affective relations between people and street dogs might play in facilitating psychosocial wellbeing requires further research.

People’s narratives about where dogs belong and how they intersect with public health concerns provide a window into the multidimensionality of people-street dog relationships. Street dogs are not just out-of-place animals that need controlling, rescuing or rehoming. They are integral inhabitants of the multispecies city, and like their human counterparts, can be involved in positive, negative and neutral interactions with their human and nonhuman cohabitants. They can be nuisances and pose health risks, but they are also vulnerable beings that are tolerated, cared for and cherished.

These understandings of free-living dogs and their place in human society are not in alignment with existing public health approaches, and could well underlie lack of political will for or inadequate public participation in dog control programmes. They also manifest as a suspicion of dog-catching for ABC-ARV (Castillo-Neyra et al., 2017; cf. Degeling et al., 2018): semistructured interviews revealed that people are not often sure of what will be done to these animals or whether they will be returned in good health, while 17% of survey respondents said that they had actively prevented dogs from being caught for ABC-ARV.

This multidimensionality is seen in the rich knowledge among pavement dwellers and waste-workers on how to live safely with dogs (sections ‘Conflict and care’ and ‘Knowledge’). We suggest that this knowledge is rooted in learning that comes from daily proximity and interactions—positive and negative—between people and dogs. In other words, it stems from everyday practices of cohabitation and the resulting attention to and engagement with street dogs as members of the public (as opposed to top-down awareness programmes on rabies and bites).

Equally, those people who are not tolerant of street dogs simply want dogs eradicated, regardless of scientific evidence about the greater efficacy of ARV-ABC. Here, even if the spectre of rabies is used by groups that call for eradication, they remain unconvinced by the science behind One Health-oriented approaches arguably because the underlying motives are different. Some do not see free-living dogs as belonging in ‘developed’ societies. Others are bothered by barking and general nuisance; yet others are concerned about threats to valued wildlife (e.g., Vanak, 2008). The stable and rabies-free street dog populations that ABC-ARV programmes aim to establish do not guarantee immediate and complete eradication of free-living dogs, and therefore do not satisfy those who hold these views. This has led to court cases asking for the repeal of the 2001 legislation and the reintroduction of killing, as well as ongoing extra-legal killing or removal of street dogs. The latter (killing and removal) undermines the ecological and epidemiological logics that ABC-ARV programmes are based on, and weakens their efficacy (Morters et al., 2013).

### Pathways to decolonisation

The key point here is that the intersections between street dogs and public health extend beyond rabies and encompass both positive and negative dimensions. The focus of current public health agendas on rabies, bites, ABC-ARV and responsible dog ownership does not sufficiently take into account this multidimensionality. There is therefore an urgent need to decolonise and expand dominant notions of both public health and healthy publics in relation to the ‘more-than-human’ (Chakrabarty, 2009; Sundberg, 2014).

In the context of rabies, the decolonisation of public health agendas involves paying attention to, without reifying or essentialising, human-dog interactions that do not conform to Western norms of dog ownership. Decolonisation involves taking seriously the plurality of interrelations between street dogs, health and people. This generates fresh, perhaps counter-intuitive, ways of thinking about and approaching the emergent possibilities of healthy publics. For instance, it might be that dismantling current modes of people-street dog cohabitation (e.g., through adoption or reduction of street dog populations) might adversely affect existing knowledge levels about safe cohabitation. There might be value in strengthening and reinforcing long-standing traditions of cohabitation through education on dog behaviour, dog ecology and conflict prevention, and better delivery of post-exposure prophylaxis instead of the heavy and often singular emphasis on interventions on dogs to control rabies.

Decolonisation reverses established conceptions of expertise. Instead of relying on awareness campaigns and public health materials designed by scientists, policy-makers and expert practitioners which are more likely to replicate transnational conceptions of dogs and public health (e.g., bite prevention educational materials are often focused on ‘owned’ dogs (WHO et al., 2019)), it emphasises engaging with the grounded knowledge on safe cohabitation found among people (such as waste-workers and pavement dwellers) who live and work in close proximity to, and in everyday interaction with, street dogs. Equally, taking into account the positive dimensions of people-street dog relationships, and ideas about the vulnerability and belonging of free-living dogs, calls for the reconfiguration of dog management programmes in ways that are more accountable to the wellbeing and ecologies of the dogs that are intervened upon. Most crucially, in taking seriously the understanding of free-living dogs as an integral part of the public, as part of the *more-than-human public*, decolonisation requires the subversion of the implicit anthropocentrism that underlies public health, including One Health, approaches.

## Conclusion

Through investigations of perceptions, knowledges and interactions vis-à-vis people-street dog relationships, we have argued for the decolonisation of public health agendas on rabies. Free-living dogs have always been a part of Indian society, of the public. What has changed over time is how they feature in public health discourse and practice. The British colonial government instituted state-managed control of dogs for reasons of human health, and along with that, the overarching idea that free-living dogs are out-of-place reservoirs of disease that need control. Despite the variations between killing and ABC-ARV as management practices, the underlying approach and understanding of street dogs as external threats to human society, to the ‘public’, have remained dominant in public health agendas. These conceptions echo, to varying degrees, how colonial governments viewed ‘native’ (human) populations in public health programmes against malaria—as vectors and reservoirs of the disease that had to be contained to preserve their capacity to work or to safeguard their colonial rulers (Harrison, 1994; Bhattacharya, 2012; Mishra, 2015; Rehman, 2019).

Our research shows that street dogs are located in people’s perceptions and everyday practices as both threats and valued cohabitants. They are seen as both risky and vulnerable, and an integral part of society. In other words, they are a constituent component of the more-than-human public. If the move from public health to healthy publics requires from the ground up understandings of health, this case of street dogs and rabies encourages a reorientation towards *decolonised healthy more-than-human publics.* In this expanded paradigm, free-living dogs would not be external vectors to be controlled, but part of a society which has human and nonhuman members who cohabit in mutual and uneven risk and vulnerability. Such a paradigm would also pay more explicit attention to historically-rooted power imbalances and (post)colonial legacies that inform and inhibit the formation of healthy publics.

The reorientation of rabies research and practice would involve moving away from the singular focus on the eradication of dog-mediated rabies to grasp the multidimensionality of relationships between people and dogs, and to examine the means and mechanisms that enable healthy more-than-human publics. It also entails bearing in mind long-standing social inequities in the pursuit of rabies elimination. Such inequities might revolve around ethnicity, class and gender, and we would argue, also around species. Tackling canine rabies has favour as the most efficient way of eliminating human rabies because of the difficulties in achieving adequate coverage of (human) post-exposure prophylaxis and rabies awareness (Cleaveland et al., 2006; Hampson et al., 2015; Lavan et al., 2017; Rock et al., 2017). In other words, it is simpler to intervene on dogs than work with people. These judgments about efficiency are ultimately rooted in the fact that issues relating to consent and harm are easily elided when it comes to nonhuman animals, i.e., they are rooted in anthropocentrism.

In practical terms, reorientation might involve:

1. Investigating the biosocial conditions under which conflict, both dog-on-human and human-on-dog, occurs so as to prevent such conflict (e.g., our research indicates that large collections of food waste can result in fighting among dogs with spill-on effects on people);
2. Drawing on people’s (local) knowledge to develop educational materials and techniques for safe cohabitation, and strengthening traditions of cohabitation through further research on the multidimensional (including positive) aspects of people-street dog relationships;
3. Building knowledge about street dog ecologies and cultures, and the rationale for vaccination/neutering programmes;
4. Strengthening awareness about and addressing inequities in reach of post-exposure prophylaxis;
5. Improving dog management programmes with information on dog movement and ecologies, and with serious attention to and research on animal wellbeing in all stages;
6. Investigating and contesting the diffusion and entrenchment of (neo)colonial agendas of rabies and street dogs.

This reorientation of rabies research and practice towards healthy more-than-human publics has broader implications. The history of zoonotic disease control is full of stories of battles against external agents (vectors, reservoirs, pathogens), followed by failure in the face of resistance (in these external agents), followed by newer and newer interventions for control, and newer and newer forms of resistance and emergent problems (Asdal et al., 2016). Technical “fixes”, such as draining swamps and deploying insecticides, have produced serious unintended health and ecological consequences (McCann, 2015; Deb Roy, 2017). Anti-microbial resistance, insecticide-resistant bugs, newly emerging and highly pathogenic infectious diseases, and ‘ecological armageddon’ are all manifestations of battle, limited success, resistance, failure, unintended consequences, and further battle (Leather, 2018). Even in the case of rabies, those regions (such as North America and Europe) that have had relative success in eliminating the disease through dog control are now seen as facing renewed threats because the rabies virus has undergone “host shifts to bats and other wild animals” (Gilbert, 2018).

These chequered histories demand fresh approaches to zoonoses built on expanded conceptions of healthy publics. Such approaches would pay more attention to transnational and local historical contexts and (neo)colonial legacies, move away from the over-reliance and singular emphasis on expert knowledge, be more circumspect about assumptions regarding the human capacity to fully know and control biosocial interactions and processes, and be founded on less anthropocentric understandings of health and the public. The challenge of street dogs and public health in India (and more widely in South Asia) promises to benefit from and provide a fertile ground to develop and strengthen decolonised paradigms of public health. In the era of global, and sometimes apocalyptic, concerns about infectious diseases, the decolonisation of rabies research and practice offers fruitful and novel pathways for reorienting public health agendas on zoonoses towards the enabling of healthy more-than-human publics.

## Supplementary Material

Supplementary tables 1-3 **Supplementary information** is available for this paper at https://doi.org/10.1057/s41599-019-0358-y.

## Figures and Tables

**Table 1:** Key responses from semi-structured interviews on how to avoid conflict.

Problem	Response	What *not* to do
Barking/growling	Stand quietly Ignore the dog Foster familiarity Avoid wearing hats (from waste-workers) Move away slowly Talk to the dog in a gentle tone	Do not make sudden or fast moves Do not hit the animal
Chasing (of vehicles)	Go slow or stop the vehicle; if in an autorickshaw, shake the vehicle a little bit Pretend to pick up a stone or a stick	
Chasing (of people)	Raise voice, pretend to throw a stone Offer biscuits/food	Do not run
General	People familiar with the animal often intervene and tell (successfully) the dog to stop barking/chasing	

**Table 2:** The percentage of survey participants who spontaneously mentioned each reason that might make street dogs act aggressively.

Reason for aggression	% of people who produced this response	95% confidence interval
When they have puppies	41%	±4.81
At night-time	34%	±4.64
When strangers enter the area	32%	±4.57
When they are hungry	30%	±4.49
When they see people running	22%	±4.05
When they see moving vehicles	20%	±3.92
When they are scared	18%	±3.76
When people are aggressive towards them	18%	±3.76
When they are in groups	12%	±3.18
That is just their nature	10%	±2.94
When they are hurt or injured	7%	±2.5
During the mating season	1%	±>0.97

## Notes

1 Nor did we observe any differences in ATSD between religious groups, though these analyses were limited by the dominance of one religious group in the sample (Hindu -83%); this reflects the demographic composition of Chennai (80.73% Hindu, 2011 Census).

2 All reported 95% Confidence Intervals are for city-wide population estimates based on our sample of 401 and an estimated Chennai population of 10 million.

3 Indeed, although the eight items used to create the ATSD scale had (just) acceptable internal consistency (Cronbach’s Alpha = 0.69), a principal component analysis (with varimax rotation) indicated three potentially separable attitudes relating to: a) fear and dislike of dogs (which also correspond with a sense of their belonging in the city), b) seeing them as a potential problem, pest or nuisance, and c) seeing them as having rights and as being harmless/vulnerable.

4 Participants are referred to by pseudonyms, gender (M/F), socio-economic status (LIG/MIG/UIG = low/middle/upper SES) or occupation, and year of interview.

5 This reflects the view that street dogs are a symbol of ‘underdevelopment’, and therefore not befitting of India and nationalistic aspirations for the country to be an ‘emerging global superpower’.

6 That is not to say that Indians uniformly condemned the culls. Some local Madras newspapers called for the use of poison rather than clubbing as a more effective and humane way to kill dogs (Government of Madras, 1889).
