# Supplementary material for: Reorienting rabies research and practice: Lessons from India
Source: Palgrave Commun. Author manuscript; Available in PMC 2020 Aug 25. (PMC7115975; doi:10.1057/s41599-019-0358-y)
Supplement: Supplementary tables 1-3 [file EMS88747-supplement-Supplementary_tables_1_3.pdf]

Reorienting rabies research and practice: Lessons from India
Supplementary Materials

Author information

Krithika Srinivasan (Corresponding author)
Institute of Geography
University of Edinburgh
K.srinivasan@ed.ac.uk

Tim Kurz
Department of Psychology
University of Bath

Pradeep Kuttuva
Independent Researcher

Chris Pearson
Department of History
University of Liverpool

Supplementary Table 1: Means and Standard Deviations of scores on each ATSD item

ATSD item	N	Minimum	Maximum	Mean	Standard Deviation
Dislike of street dogs	401	1.00	5.00	3.1646	1.26010
Fear of street dogs	401	1.00	5.00	3.2594	1.29906
Street dogs are a problem	401	1.00	5.00	3.6434	1.02957
Street dogs do not belong in Chennai	401	1.00	5.00	2.7556	1.17479
Street dogs are pests	401	1.00	5.00	3.6409	.99786
Street dogs do not have a right to live on the streets	401	1.00	5.00	2.2868	.91654
Street dogs are a nuisance	401	1.00	5.00	3.6035	1.02708
Street dogs are not harmless/vulnerable	401	1.00	5.00	2.2219	.92904

Supplementary Table 2: The percentage of the sample of survey participants who spontaneously mentioned each type of option in response to being asked what they thought might be the best thing to do if a dog chases you while you are riding a bicycle or motorbike.

**Response**	**% of sample who mentioned it**
Avoid riding too fast	42%
Stop the bike	41%
Avoid braking suddenly	27%
Carry and use stones and sticks	24%
Put up your legs and continue riding	19%
Avoid neighbourhoods where there are chasing dogs	20%
Avoid making weird noises	16%
Become friendly with the dog(s)	10%
Offer food to the dog(s)	9%
Talk to the dog(s)	5%
Do not make eye contact	4%
I don’t know	1.7%

Supplementary Table 3: The percentage of the sample of survey participants who spontaneously mentioned each type of action as being a good thing to do if bitten by a dog

Action	% of sample who mentioned it
Go to the doctor/hospital	81%
Wash it with soap and water	47%
Observe the dog	28%
Find out if the dog has been vaccinated	9%
Seek Ayurvedic treatment	6%
Apply some form of herb or spice	4%
Magico-religious treatment (faith healing, witchcraft etc)	3%
Apply chunaambu (slaked lime)	2%
Apply kerosense	1%
Do nothing	1%
